# Palliative Radiotherapy with or without Additional Care by a Multidisciplinary Palliative Care Team: A Retrospective Comparison

**DOI:** 10.1155/2014/715396

**Published:** 2014-03-30

**Authors:** Carsten Nieder, Kent Angelo, Astrid Dalhaug, Adam Pawinski, Gro Aandahl, Ellinor Haukland, Kirsten Engljähringer

**Affiliations:** ^1^Department of Oncology and Palliative Medicine, Nordland Hospital, 8092 Bodø, Norway; ^2^Institute of Clinical Medicine, Faculty of Health Sciences, University of Tromsø, 9037 Tromsø, Norway

## Abstract

*Purpose*. To analyze pattern of care and survival after palliative radiotherapy (RT) in patients managed exclusively by regular oncology staff or a multidisciplinary palliative care team (MPCT) in addition. *Methods*. Retrospective analysis of 522 RT courses. Comparison of Two Groups: MPCT versus none. *Results*. We analyzed 140 RT courses (27%) with MPCT care and 382 without it. The following statistically significant differences were observed: 33% of female patients had MPCT care versus only 23% of male patients and 37% of patients <65 years had MPCT care versus only 22% of older patients. MPCT patients were more likely to have poor performance status and liver metastases. In the MPCT group steroid and opioid use was significantly more common. Dose-fractionation regimens were similar. Median survival was significantly shorter in the MPCT group, 3.9 versus 6.9 months. In multivariate analysis, MPCT care was not associated with survival. Adjusted for confounders, MPCT care reduced the likelihood of incomplete RT by 33%, *P* > 0.05. *Conclusions*. Patterns of referral and care differed, for example, regarding age and medication use. It seems possible that MPCT care reduces likelihood of incomplete RT. Therefore, the impact of MPCT care on symptom control should be investigated and objective referral criteria should be developed.

## 1. Background

Patients with cancer referred to palliative radiotherapy often experience considerable burden from disease-related symptoms such as pain, bleeding, or neurological deficits, which may improve after successful completion of treatment [1]. Due to high rates of symptom improvement, radiotherapy has become a mainstay in multidisciplinary care for patients with incurable cancer. However, large variations exist in radiotherapy regimens, supportive therapy, resource utilization, involvement of other medical disciplines and professions, and care setting [2]. Several studies have shown that patients with noncurable metastatic cancer might receive too aggressive and long-standing treatment during the last months of life [3–7]. The focus on optimal supportive care may be lost or prioritized too late.

Recently, a randomized trial of early palliative care for patients with newly diagnosed metastatic non-small cell lung cancer (NSCLC), which recruited patients in the time period between 2006 and 2009, was published [8, 9]. This single institution trial included 151 patients. Early palliative care integrated with standard oncology care was compared to standard oncology care alone. Patients assigned to the experimental arm consulted with a member of the palliative care team within 3 weeks of enrollment and at least monthly thereafter. Those assigned to the standard care arm only met with the palliative care team on request from the patient, family, or oncologist. Early palliative care integrated with standard oncologic care led to significant improvements in quality of life (QoL) and mood from baseline to 12 weeks. Fewer patients received aggressive end-of-life care, yet median survival was longer among patients receiving early palliative care (11.6 versus 8.9 months). In line with previously shown improvements resulting from multidisciplinary cancer therapy (chemoradiation and surgery plus adjuvant radio- and/or chemotherapy), the study suggests that additional expertise and intervention may be beneficial in several ways, possibly even in terms of longer survival.

Based on these considerations and because a multidisciplinary palliative care team (MPCT) has been integrated in our oncology department for several years, we were interested in analyzing the impact of a MPCT on survival after palliative radiotherapy, use of radiotherapy near the end of life, and successful completion of fractionated treatment. Furthermore, we wanted to study possible imbalances regarding referral of radiotherapy patients to our MPCT.

## 2. Methods

We retrospectively reviewed the records of 412 consecutive patients with metastatic or otherwise incurable cancer receiving palliative radiotherapy at a single hospital with a dedicated palliative radiotherapy unit, Nordland Hospital Bodø, Norway (an academic teaching hospital, which is the only provider of radiation oncology services in the county of Nordland). The patients started their treatment in the time period from June 20, 2007 (date of opening of the radiotherapy unit), to December 31, 2009. A total of 579 radiotherapy courses were studied, meaning that individual patients could have received more than one course, but only 522 of these could be included because the others had incomplete medical records, which did not allow for firm assignment to one of the two study groups (MPCT or no MPCT). Referral to MPCT was not standardized. Rather, individual decisions were made by the treating clinical oncologists responsible for radiotherapy delivery, based on symptom severity, pain control, or need for initiation of home care services, taking into account patient preferences. Our MPCT staff, which collaborates closely with primary health care facilities, family doctors, and home care providers in the region, includes several professions: physician, nurse, psychologist, physiotherapist, nutritionist, and priest. Regular weekly meetings between clinical oncologists and MPCT took place. Both MPCT and radiotherapy unit operated every workday. Time from referral to MPCT to first appointment was 1-2 days. All patients were covered by the national public insurance system. Therefore, no out-of-pocket costs were incurred for any patient regardless of management approach/treatment intensity. In other words, no particular barriers prevented patients from access to our MPCT.

Radiation treatment details and date of death were available from the hospital's electronic patient record (EPR) system. The survival status and date of death or last followup of the patients were obtained from the EPR. Patients who were lost to followup were censored on the date of their last documented contact. Patients who started a new course of radiotherapy after their first one were censored on day 1 of the new course. Median followup for all censored patients was 207 days. Survival time was measured from day 1 of radiotherapy. Actuarial survival curves were generated by Kaplan Meier method and compared by log-rank test. We used IBM SPSS Statistics 20 (IBM Corporation, Armonk, NY, USA) to evaluate the association between potential predictive factors, including but not limited to eastern cooperative oncology group (ECOG) performance status (PS), age and disease extent, and referral to MPCT or other endpoints. Univariate analysis consisted of Pearson chi-square and Fisher's exact test. Factors achieving statistical significance (defined as *P* < 0.05 throughout this study in two-sided tests) were entered into multivariate analysis (logistic regression; for overall survival Cox regression).

## 3. Results

The MPCT was involved in 140 radiotherapy courses (27%); these patients were significantly younger (median age 67 versus 70.5 years, *P* = 0.001). The median time from first cancer diagnosis to radiotherapy did not differ significantly (28 versus 30 months, *P* = 0.77). In patients with metastatic cancer, median time from diagnosis of metastases to radiotherapy was similar (4 versus 6 months, *P* = 0.45).  Table 1 shows detailed information on all radiotherapy courses administered to both groups. The following statistically significant differences were observed: 33% of female patients had MPCT care versus only 23% of male patients (*P* = 0.02), and 37% of patients <65 years had MPCT care versus only 22% of older patients (*P* = 0.006). Moreover, the MPCT group contained significantly more patients with ECOG performance status 3 or 4, liver metastases, and previous systemic therapy before RT. In the MPCT group steroid use was significantly more common (57 versus 37%, *P* = 0.0001), opioid analgetic use was more common (68 versus 48%, *P* = 0.0001), and more patients were irradiated to more than one target volume (42 versus 29%, *P* = 0.01). Dose-fractionation regimens were similar.

Median survival was significantly shorter in the MPCT group, 3.9 versus 6.9 months, *P* = 0.0001. However, rate of early death within 1 month from start of radiotherapy was only numerically higher (11 versus 9%, *P* = 0.40). In multivariate Cox regression analysis, MPCT care was not associated with survival (*P* = 0.68; significant factors were ECOG performance status, liver metastases, progressive disease outside radiotherapy target volume(s) (all *P* = 0.0001), brain metastases (*P* = 0.001), and low hemoglobin (*P* = 0.018)). Failure to complete radiotherapy was similarly uncommon in both groups (6 versus 5%, *P* = 0.63). Adjusted for ECOG performance status (the only significant predictor of incomplete radiotherapy in multivariate logistic regression analysis, *P* = 0.01), MPCT care reduced the likelihood of incomplete radiotherapy by 33%. However, this difference was not statistically significant (few events and low statistical power).

## 4. Discussion

In recent years, the impact of multidisciplinary cancer care has been recognized widely. MPCTs have been established in many hospitals with radiotherapy facilities, and such teams often provide additional supportive care interventions [10], physical exercise and therapy [11], and spiritual care, focusing on patients and caregivers [12], but their influence on pattern of care and outcomes has not been studied extensively. Pituskin et al. reported on multidisciplinary assessment of patients with symptomatic bone metastases attending a dedicated outpatient palliative radiotherapy clinic [13]. Consecutive patients were screened for symptoms and needs relevant to their medications, nutritional intake, activities of daily living, and psychosocial and spiritual concerns from January 1 to December 31, 2007. Consultations by appropriate team members and resulting recommendations were collected prospectively. Patients who received radiotherapy were contacted by telephone four weeks later to assess symptom outcomes. A total of 106 clinic visits by 82 individual patients occurred. In addition to pain relief, significant improvements in tiredness, depression, anxiety, drowsiness, and overall well-being were reported at four weeks. Temel et al. reported that early palliative care integrated with standard oncology care was superior to standard oncology care alone (not limited to radiotherapy) in patients with NSCLC [8]. Their randomized study is inspiring in several ways, including an unexpected difference in overall survival. The latter was not the primary endpoint of the study and could result from imbalances in prognostic factors and the limited study size. However, it stimulates further research and also prompted our group to perform the analyses discussed here.

On the one hand, our study was comprehensive, with access to many baseline parameters, and included patients from a representative 2.5-year time period. On the other hand, MPCT referral was not standardized and was limited to 27% of all radiotherapy courses. In other words, group size was still not optimal. As might be the case with most retrospective studies, not all data were available from the patients' records, and heterogeneity regarding radiotherapy targets existed. Symptom severity and improvement were not assessed. Hidden sources of bias cannot be excluded. Nevertheless, interesting findings, which might stimulate larger studies, emerged. As expected intuitively, patients with simultaneous MPCT care had more advanced disease (higher rates of liver metastases, systemic therapy, and more than one irradiated site) and poorer performance status, suggesting a higher symptom burden and need for supportive measures. On the contrary, the observed gender and age differences are unexpected and could reflect biased referral decisions, even if no obvious barriers such as insurance status, copayments, or other financial burdens existed. More standardized referral criteria need to be developed, based, for example, on patient questionnaires and symptom scales such as the Edmonton Symptom Assessment System (ESAS), which is now used in our department. It is both understandable and reassuring to see that patients managed by MPCT actually received more intense analgesic treatment. Prescription of steroids might also improve QoL and reduce cancer-related fatigue [14] and was more frequent in the MPCT group. As already mentioned, we were not able to retrospectively collect information on patient satisfaction or QoL. It is therefore unknown whether patients who were not in contact with our MPCT had good or unsatisfactory symptom control and what should be considered an optimal referral or utilization rate.

MPCT care had no significant influence on overall survival or radiotherapy utilization near the end of life. In contrast to the randomized study by Temel et al. [8], most of our patients received late rather than early palliative care. At this stage of disease, considerable survival improvements are no longer realistic. The aim and primary endpoint of the randomized NSCLC study was not to detect improved survival, and there could be other explanations why a rather small randomized trial shows an apparent survival advantage for one of the study arms. However, other data suggest that patients with metastatic NSCLC and impaired QoL had shorter survival than those with better QoL [15]. So if early palliative care enhances QoL, for example, by providing psychosocial support (actually not all patients have sufficient network of family and friends), better symptom control, and fewer treatment complications, survival might increase. In our study, few patients were unable to complete their prescribed course of radiotherapy, probably because hypofractionated regimens tailored to the expected prognosis were commonly used. Given the adverse impact of performance status in the MPCT group, the high rate of radiotherapy completion is noteworthy. It appears possible that MPCTs also can contribute to management of typical radiotherapy-induced side effects, for example, nausea and vomiting [16], thereby facilitating administration of all fractions according to schedule. Adjusted multivariate analysis suggested that MPCT care may contribute to successful treatment completion, although differences were not statistically significant. Much larger studies are necessary to confirm this hypothesis with sufficient statistical power. It is tempting to speculate that patients who complete radiotherapy are more likely to experience its full benefit. If true, MPCT contributions might add even more value than previously thought.

## 5. Conclusions

Increasing evidence suggests that MPCTs play an important role in the multidisciplinary management of patients with incurable cancer. Our data provide starting points for larger prospective evaluations in the context of palliative radiotherapy.

## Figures and Tables

**Table 1:** Patient characteristics.

Univariate analysis of baseline parameters for patients' individual RT courses administered with or without care by multidisciplinary palliative care team (MPCT)			
Parameter	Without MPCT number of RT courses (%)	With MPCT number of RT courses (%)	*P* value
Patient number	382	140	
ECOG performance status			
0	60 (16)	9 (6)	
1	112 (29)	30 (21)	
2	116 (30)	50 (36)	
3	82 (21)	45 (32)	
4	12 (3)	6 (4)	0.004
Family^a^			
Single	102 (27)	48 (34)	
Married	212 (55)	73 (52)	
Partner	26 (7)	15 (11)	0.185
Age at RT (years)			
<65	104 (27)	60 (43)	
65–69	70 (18)	20 (14)	
70–74	52 (14)	22 (16)	
75–79	86 (23)	20 (14)	
≥80	70 (18)	18 (13)	0.006
Gender			
Male	242 (63)	73 (52)	
Female	139 (36)	67 (48)	0.02
Primary tumor site			
Prostate	96 (25)	29 (21)	
Breast	38 (10)	24 (17)	
Lung (small cell)	23 (6)	7 (5)	
Lung (non-small cell)	70 (18)	21 (15)	
Colorectal	24 (6)	11 (8)	
Kidney	23 (6)	8 (6)	
Bladder	25 (7)	5 (4)	
Malignant melanoma	12 (3)	2 (1)	
Other	71 (19)	33 (24)	0.39
Incomplete RT^b^			
No	341 (96)	114 (94)	
Yes	16 (4)	7 (6)	0.63
Total number of TV in RT course			
1	272 (71)	81 (58)	
2	90 (24)	46 (33)	
3	20 (5)	13 (9)	0.01
Number of RT fractions			
Single	41 (11)	19 (14)	
2–10	249 (65)	92 (66)	
>10	92 (24)	29 (21)	0.46
Dose per fraction (Gy)			
<3	44 (12)	13 (9)	
3	190 (50)	64 (46)	
>3	148 (39)	63 (45)	0.41
Selected target types			
Bone metastases	240 (63)	95 (68)	0.35
Brain metastases	58 (15)	29 (21)	0.15
Known liver metastases^a^			
No	311 (81)	103 (74)	
Yes	69 (18)	37 (26)	0.049
Known lung metastases^a^			
No	304	108	
Yes	76	32	0.47
Known adrenal gland metastases^a^			
No	346	127	
Yes	34	13	0.87
Metastatic disease^a^			
No	51 (13)	13 (9)	
Yes	303 (79)	116 (83)	0.23
Progressive disease outside TV^a^			
No	147 (38)	48 (34)	
Yes	225 (59)	86 (61)	0.47
Hemoglobin before RT^a^			
Low	230 (60)	97 (69)	
Normal	116 (30)	39 (28)	0.33
Steroids during RT^a,c^			
No	183 (48)	48 (34)	
Yes	141 (37)	80 (57)	0.0001
Analgetics^a,c^			
No opioids	158 (41)	34 (24)	
Oral/transdermal opioids	174 (46)	80 (57)	
Continuous opioids (pump)	6 (2)	15 (11)	0.0001
Systemic cancer treatment^a^			
No	186 (49)	48 (34)	
Before RT	173 (45)	80 (57)	0.006

RT: radiotherapy, TV: target volume. ^a^Missing information in some cases; ^b^excluding single fraction RT, which was always completed; ^c^information collected from patient charts rather than pharmacy databases.
